# Disease Susceptibility to ST11 Complex Meningococci Bearing Serogroup C or W135 Polysaccharide Capsules, North America^1^

**DOI:** 10.3201/eid1010.040335

**Published:** 2004-10

**Authors:** Andrew J. Pollard, Jan Ochnio, Margaret Ho, Martin Callaghan, Mark Bigham, Simon Dobson

**Affiliations:** * University of Oxford, Oxford, United Kingdom;; † University of British Columbia, Vancouver, Canada;; ‡ Canadian Blood Services, Vancouver, Canada

**Keywords:** Neisseria meningitides, population immunity, meningococcal disease, research

## Abstract

Clusters of meningococcal disease caused by a hyperinvasive lineage of *Neisseria meningitidis*, the ST11 complex, bearing a serogroup C polysaccharide capsule, have been prominent in Europe and North America since the early 1990s. This situation has led to expensive public health measures for outbreak control and, finally, to the introduction of a serogroup C glyconjugate vaccine into the primary immunization schedule in the United Kingdom and elsewhere. ST11 complex meningococci may also express serogroup W135 polysaccharide capsules. We investigated the level of population immunity to this hyperinvasive clone in association with the appearance of outbreaks of meningococcal disease in southern British Columbia. We found that most adults and almost all children were apparently susceptible to infection with ST11 complex meningococci bearing both C and W135 polysaccharide capsules, which suggests that a vaccine program directed against only serogroup C meningococci may be insufficient to prevent hyperinvasive ST11 disease.

## Abstract 2

Study population was susceptible to ST11 complex meningococci bearing both C and W135 polysaccharide capsules; vaccine against serogroup C meningococci may not prevent ST11 disease.

In 1919, George Heist and co-workers established that clotted blood from different persons varied in its ability to kill *Neisseria meningitidis* in a capillary tube. When Heist, whose blood had no bactericidal activity, acquired *N. meningitidis* infection, the link between serum bactericidal activity and resistance to meningococcal infection was proven (*1*). Nearly half a century later, Goldschneider et al. found that <20% of infants 1 year of age had anti–serogroup C meningococcal bactericidal activity in their blood, but >60% of teenagers and 75% of adults had protective titers (*2*). Disease rates were inversely related to the population bactericidal titers, with high rates of disease in young children and low rates in adults.

Groups of genetically related meningococci can be identified by using the electrophoretic mobility of cytoplasmic proteins (electrophoretic type; ET) or by nucleotide sequencing of "housekeeping" genes (sequence type; ST). During the 1990s, a clone of serogroup C *N. meningitidis* (ET-37 complex; ST11 complex) was responsible for outbreaks of meningococcal disease in the United States, Canada, and Europe, predominantly affecting teenagers and young adults and leading to repeated and massive public health interventions (*3*). In 1999, disease attributed to this clone led to serogroup C glyconjugate vaccine's introduction into the primary immunization schedule in the United Kingdom (*4*). From December 2000 to April 2001, a cluster of seven cases of invasive serogroup C meningococcal infection occurred in a community of 120,000 in southern British Columbia, Canada; five of the seven cases were in persons 18–27 years of age, which raises the possibility that more susceptible persons were found in this population than were previously inferred from data described by Goldschneider et al. 30 years ago (*2*).

During the 1990s, most ST11 complex *N. meningitidis* isolates in Canada bore an α2-9 N-acetyl neuraminic acid (serogroup C) capsule, but recent epidemics of meningococcal disease, particularly in Africa, have been associated with ST11 meningococci bearing the W135 capsule. This finding suggests that this hyperinvasive lineage might also spread to populations with low levels of population immunity against W135 capsule-bearing organisms. In addition, ST11 meningococci bearing serogroup B, and occasionally Y capsules, also occur (*5*). We examined population immunity to ST11 complex meningococci bearing serogroup C or W135 polysaccharide capsules.

## Methods

**Figure 1:**
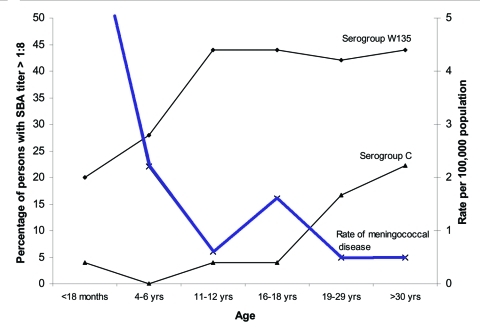
Incidence (cases/100,000/year) of meningococcal disease (average rates 1985–2000) in relation to serogroup C and W135 bactericidal antibody titers in British Columbia against a local ST11 outbreak isolate (AOBZ1379) and Z1582/FC978 (a Canadian clinical isolate from 2000 bearing the W135 capsule), respectively.

We obtained serum specimens from 175 healthy persons from southern British Columbia (Table) after the study protocol was reviewed by the University of British Columbia Clinical Research Ethics Board. Using these serum specimens, we examined the SBA, the ability of serum to kill meningococci when mixed with exogenous complement, against three target strains of *N. meningitidis*, AOBZ1379(c) (the outbreak clinical isolate from British Columbia; C:2a,P1.5; ET15), Z1582/FC978 (a Canadian clinical isolate from 2000 bearing the W135 capsule, W135:2a:P1.5,2) and C11 (60E; C:16:P1.7-1,1, a standard reference strain [*2*]), according to standard methods by using baby rabbit complement (Pel-Freeze Inc., Rodgerson, AR) as the exogenous source of complement (*6*). The highest serum concentration tested in the assay was 1:4, and a titer of 1:2 was assigned to sera with <50% killing at this concentration. The three bacterial strains were characterized by multilocus sequence typing, which confirmed the sequence-types as ST11, ST11, and ST345, respectively. We defined protection against serogroup C *N. meningitidis* as a serum bactericidal titer of >1:8 (*4**,**7*). Incidence data for rates of meningococcal disease were obtained from the British Columbia Center for Disease Control, Vancouver, Canada (Figure 1). The data are reported as geometric mean titers (GMTs) and as a percentage of the population higher than the protective threshold.

**Table:** Serum bactericidal activity against target strains of *Neisseria meningitidis*^a^ for serum samples from different age groups^b^

Age	Serogroup C (ST11)				Serogroup W135 (ST11)			
	n	GMSBAT	95% CI	% >1:8	n	GMSBAT	95% CI	% >1:8
2 mo	24	2.4	2.3–2.7	4				
18 mo	25	2.1	2.1–2.3	4	25	4.3	3.8–5.0	20
4–6 y	25	2	2.0–2.0	0	25	8	6.6–9.7	28
11–12 y	25	2.4	2.2–2.5	4	25	16.5	13.4–20.1	44
16–18 y	25	2.3	2.2–2.4	4	25	24.3	19.4–30.3	44
19–29 y	24	4	3.5–4.6	17	7	21.5	8.1–57.2	43
>30 y	27	7	5.8–8.6	22	18	19.3	13.8–27.3	44

^a^AOBZ1379(c) (the outbreak clinical isolate from British Columbia) or Z1582/FC978 (a Canadian clinical isolate from 2000 bearing the W135 capsule). ^b^GMSBAT, geometric mean serum bactericidal assay titers; CI, confidence interval.

## Results

**Figure 2:**
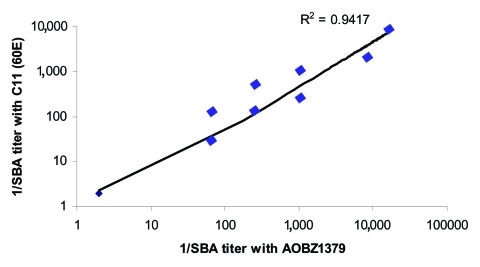
Correlation between bactericidal titers against an outbreak strain of serogroup C meningococcus (AOBZ1379) from southern British Columbia and the standard reference strain C11 from 27 serum specimens.

The GMT of the bactericidal antibody against the outbreak strain of serogroup C, ST11 complex *N. meningitidis* was 1:2 in serum specimens from all children <18 years of age who were studied. In the group 19–29 years of age, the GMT rose to 1:4 and reached 1:7 in adults >30 years of age (Table). Three percent of children <18 years, and 19% of adults >19 years of age (median age 33 years), had serum bactericidal titers above or equal to the "protective" level (1:8) against this outbreak strain (Figure 1, Table). These data correlated closely with titers obtained by using strain C11, the standard reference strain used in serogroup C bactericidal assays (Figure 2). The reference serum CDC1992 (National Institutes for Biological Standards and Control) also produced identical SBA titers with both meningococcal strains.

The GMT of bactericidal antibody against the W135 strain of *N. meningitidis* rose steadily from 1:4 in those 18 months of age to 1:8, 1:16, 1:24, and 1:20 in those aged 4–6 years, 11–12 years, 16–18 years, and >19 years, respectively (Table). Sera tested from 20% of infants had bactericidal activity against the serogroup W135 ST11 complex organism, with titers >1:8 in up to 40% of serum samples from persons in the second decade of life and 44% in serum from adults. Rates of meningococcal disease in the population were inversely related to the population levels of bactericidal antibody for both serogroup C and serogroup W135 cases (Figure 1, Table).

## Conclusions

This study suggests that population immunity against hyperinvasive lineages of meningococci are low in a North American population. Protective serum bactericidal titers were present in 3% of children <18 years of age and 19% of adults (median age 33) against the outbreak strain of serogroup C, ST11 complex *N. meningitidis* (Figure 1). These bactericidal titers were low even though we used a complement source (baby rabbit serum) that is associated with higher SBA titers than the human complement used in Goldschneider et al.'s study in the 1960s, which found considerably higher levels of protection (*2*). Of note, 40% of meningococcal disease in the 1990s in Canada was caused by serogroup C meningococci (*8*). To exclude the possibility that the outbreak strain was more resistant to serum bactericidal activity, we compared these data with those obtained by using C11, the serogroup C meningococcus used in 1969 by Goldschneider et al.; however, we found a close correlation (r = 0.97) between strains, which indicates no differences in serum resistance attributable to the different strains (Figure 2). Since these strains belong to different sequence types and they carry different subcapsular outer membrane proteins (C:16:P1.7-1,1 versus C:2a:P1.5), these data may indicate that antibodies against their common antigen (the serogroup C capsule) are more important than subcapsular antigens in the SBA. However, other subcapsular proteins common to both isolates may not have been identified, which could be responsible for this observation. By contrast, no correlation was seen (r = 0.2) between SBA titers achieved with 60E and Z1582/FC978, the ST11 W135 isolate, even though both isolates shared the same major outer membrane proteins (C:2a:P1.5); this finding further supports the importance of anticapsular functional antibodies in this assay.

Before routine immunization in the United Kingdom with serogroup C glyconjugate meningococcal vaccine was begun, Trotter et al. found bactericidal titers >1:8 in 10% to 20% of infants and 25% of adults (*9*). Similarly, Jones et al., found that 10% of university students appeared to have protective serum bactericidal levels (*10*). These U.K. findings are similar to our Canadian data that suggest that population immunity against serogroup C meningococcus may now be lower than previously described in countries without an immunization program.

We also found that 20% of infants had serum bactericidal activity against the serogroup W135 ST11 complex organism, with titers >1:8 in up to 40% in serum specimens from persons in the second decade of life and 44% in serum specimens from adults. The importance of anti-serogroup W135 serum bactericidal titers for protection is not well defined, but, extrapolating from serogroup C data, our results may indicate that 60%–80% of persons are susceptible to W135 disease. Less than 5% of laboratory-confirmed cases of invasive meningococcal disease in British Columbia are attributable to serogroup W135, but epidemic disease caused by ST11 complex organisms that bear the W135 capsule has been recognized in recent years and found to be associated with travel (*11*) and sub-Saharan African populations (*12*). Meningococci bearing the W135 polysaccharide capsule have been a relatively infrequent cause of sporadic cases of meningococcal disease in the 30 years since the first descriptions of this serogroup from cases in the U.S. army (*13**,**14*). However, in 2000 and 2001, an outbreak of disease occurred among pilgrims traveling to Mecca in Saudi Arabia for the annual hajj pilgrimage (*14**,**15*). This outbreak was caused by serogroup W135 meningococci from the ST11 (ET-37) complex (*16*), which was previously associated with hyperinvasive serogroup C disease. Since 2000, ST11 complex serogroup W135 meningococci have also appeared in sub-Saharan Africa and caused large epidemics (*17*). The association of epidemics of disease with meningococci of the hyperinvasive ST11 lineage that bears the W135 capsule is a cause for concern for populations with limited population immunity to these meningococci.

Figure 1 shows an apparent paradox: although the disease rate is decreasing substantially in persons <18 months to 11–12 years, little serologic evidence exists of a parallel rise in protection, i.e., the geometric mean SBA titers are almost constant, and the percentage of children with SBA titers <1:8 in all age groups up to those 16–18 years remains low. This finding could indicate that SBA lacks sensitivity (perhaps protection occurs before a titer of 1:8 is reached) or that protection rises through immune mechanisms other than the bactericidal combination of antibody and complement (such as osponophagocytosis). A decrease in exposure to serogroup C meningococci or a reduction in other cofactors that lead to invasive disease may also occur in this age group.

ST11 lineage meningococci may also bear serogroup B capsules, and evidence exists that a switch between serogroups may occur naturally in populations (*18**,**19*), perhaps a process that is favored by population immunity. Whether immunization with vaccines that target serogroup C capsule–bearing ST11 complex meningococci will favor the evolution of B or W135 ST11 complex bacteria in the next few years is not clear. No evidence of the emergence of serogroup B ST11 complex meningococci has been reported in the United Kingdom since the serogroup C glyconjugate vaccine was introduced in 1999. However, in Spain, ST11 serogroup B meningococci have emerged since vaccine introduction, which raises the possibility that, in some circumstances, immunologic pressure on meningococcal populations by vaccine may lead to capsule switching (*20*).

Recent data after the serogroup C glyconjugate vaccine was implemented in the United Kingdom strongly suggest that SBA titers >1:8 are required for protection against serogroup C meningococcal disease (*4**,**7*). Data presented here indicate that a high proportion of adults, and almost all children, may be susceptible to disease caused by the hyperinvasive ST11 clone of serogroup C lineage that is prevalent in North America. These findings support the value of the childhood immunization program with serogroup C meningococcal glyconjugate vaccines, implemented in British Columbia, Canada, since September 2003. However, the propensity of this clone to express capsular polysaccharides other than serogroup C, including W135, and the apparently low population immunity for bacteria bearing the W135 capsule suggest that monovalent serogroup C vaccines may be insufficient to control this widely distributed hyperinvasive lineage of meningococcus, the ST11 complex.
